# Identification of Key Functions Required for Production and Utilization of the Siderophore Piscibactin Encoded by the High-Pathogenicity Island *irp*-HPI in *Vibrionaceae*

**DOI:** 10.3390/ijms23168865

**Published:** 2022-08-09

**Authors:** Marta A. Lages, Lucía Ageitos, Jaime Rodríguez, Carlos Jiménez, Manuel L. Lemos, Miguel Balado

**Affiliations:** 1 Departmento de Microbiología y Parasitología, Instituto de Acuicultura, Universidade de Santiago de Compostela, 15782 Santiago de Compostela, Spain; 2 Centro de Investigacións Científicas Avanzadas (CICA), Departamento de Química, Facultade de Ciencias, Universidade da Coruña, 15071 A Coruña, Spain

**Keywords:** siderophores, piscibactin, *Vibrio anguillarum*, *Vibrionaceae*, bacterial fish diseases

## Abstract

Piscibactin is a widespread siderophore system present in many different bacteria, especially within the *Vibrionaceae* family. Previous works showed that most functions required for biosynthesis and transport of this siderophore are encoded by the high-pathogenicity island *irp*-HPI. In the present work, using *Vibrio anguillarum* as a model, we could identify additional key functions encoded by *irp*-HPI that are necessary for piscibactin production and transport and that have remained unknown. Allelic exchange mutagenesis, combined with cross-feeding bioassays and LC-MS analysis, were used to demonstrate that Irp4 protein is an essential component for piscibactin synthesis since it is the thioesterase required for nascent piscibactin be released from the NRPS Irp1. We also show that Irp8 is a MFS-type protein essential for piscibactin secretion. In addition, after passage through the outer membrane transporter FrpA, the completion of ferri-piscibactin internalization through the inner membrane would be achieved by the ABC-type transporter FrpBC. The expression of this transporter is coordinated with the expression of FrpA and with the genes encoding biosynthetic functions. Since piscibactin is a major virulence factor of some pathogenic vibrios, the elements of biosynthesis and transport described here could be additional interesting targets for the design of novel antimicrobials against these bacterial pathogens.

## 1. Introduction

**Figure 1:**
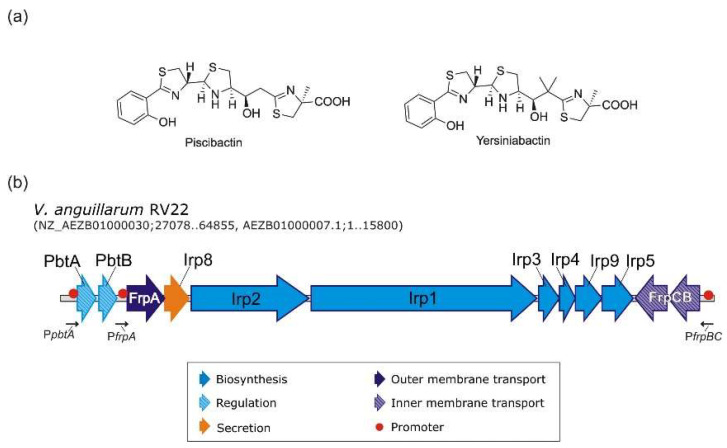
(**a**) Chemical structures of the siderophores piscibactin and yersiniabactin; (**b**) genetic map of irp gene cluster encoding piscibactin system in *Vibrio anguillarum* [7].

Iron is essential for the survival and growth of almost all organisms, but its availability is limited in the environment and within the host fluids [1]. In response to iron starvation, bacterial pathogens have developed specific and sophisticated iron uptake mechanisms such as the use of siderophores [2]. There are hundreds of different siderophores with unique chemical structures, although most of them can be grouped in a few classes according to the Fe(III) binding functional group [3]. Piscibactin is a phenolate siderophore made from salycilate and three Cys residues. Its chemical structure is closely related to yersiniabactin (the siderophore of *Yersinia pestis* and *Y. enterocolitica*), from which piscibactin differs by the lack of two geminal methyl groups [4] (Figure 1a).

The synthesis and utilization of piscibactin is encoded by a High-Pathogenicity Genomic Island named *irp*-HPI, with high homology to the *Yersinia* spp. HPI encoding yersiniabactin [5,6,7] (Figure 1). The *irp*-HPI is mainly present in species of the *Vibrionaceae* family, and it has been demonstrated to constitute a major virulence factor of certain worldwide important bacterial fish pathogens such as *Photobacterium damselae* subsp. *piscicida* [6], *Vibrio anguillarum* [7] or *V. ordalii* [8]. Piscibactin production is also necessary for full virulence of the bivalve mollusks pathogen *V. neptunius,* and *irp*-HPI is present in the genome of other mollusks *Vibrio* pathogens of the Coralliilyticus clade [9]. Furthermore, *irp*-HPI is widespread among many other *Vibrio* spp., including human pathogens like *V. cholerae* [10]. In addition, some other gamma-proteobacteria outside *Vibrionaceae* family also have gene clusters with homology to those contained in *irp*-HPI. This is the case of the genus *Marinomonas*, *Shewanella* [11] or the entomopathogenic nematodes symbiotic bacteria *Xenorhabdus* and *Photorhabdus*, in which the synthesis of piscibactin was recently reported [12].

Most siderophores are synthetized by multimodular non-ribosomal peptide synthetases (NRPSs) and polyketide synthases (PKS) from the assembly of different amino acid and polyketide moieties, respectively. Each module of a NRPS/PKS incorporates a single precursor into the nascent product that will remain covalently attached to the peptidyl carrier protein (PCP) domain [13]. Thus, the final product must be released from the final PCP by a C-terminal type I thioesterase (TE) domain present in the last NRPS/PKS of the biosynthetic pathway [14]. The *irp*-HPI genomic element must encode most of the functions (proteins Irp123459) related to piscibactin biosynthesis [6,7] and regulation (PbtAB) [15], excepting a phosphopantetheinyl transferase (EntD) required for NRPS activation, whose function must be complemented in trans by an EntD homologue encoded outside *irp*-HPI genomic island [6,7]. A piscibactin synthesis pathway was proposed based on the domain organization of the biosynthetic proteins Irp123459 [4]. However, a main aspect related to piscibactin synthesis that remains unknown is the release of the siderophore from the multienzymatic complex. Piscibactin chemical structure corresponds to the premature release of the nascent siderophore from the NRPS Irp1, and the protein Irp4 was proposed as the external TE that would mediate piscibactin liberation [4]. This hypothesis has not yet been experimentally proven.

Upon synthesis, siderophores must be exported to the extracellular environment where they bind iron(III) [15]. Then, the ferri-siderophore complex must be acquired through specific transporters [1]. The genomic island *irp*-HPI could encode a putative major facilitator superfamily (MFS)-like transporter that would mediate piscibactin secretion (Irp8), a TonB-dependent outer membrane transporter (TBDT) (FrpA), and a probable ABC transporter (FrpBC) that would mediate the final ferri-siderophore internalization [5,7]. Recent work demonstrated that FrpA is the TBDT required for ferri-piscibactin utilization [16], but the actual role of Irp8 and FrpBC has not been studied so far.

Although not all *V. anguillarum* strains produce piscibactin [7], a piscibactin-producing strain of *V. anguillarum* was used as a model to study the above-described functions related to piscibactin synthesis, secretion and utilization encoded by *irp*-HPI. The generation of single defective mutants of either *irp4* or *irp8* was used to study their role in piscibactin production, whereas the mutant *frpBC* was used to define the ferri-piscibactin internalization route. The results showed that Irp4 and Irp8 are indeed required for piscibactin production and that FrpBC inactivation disables ferri-piscibactin utilization as an iron source. In addition, the transcriptional analysis of *frpBC* revealed a temperature-dependent expression under the control of the transcriptional activator PbtA. Based on the results found in this work, together with previous findings, a model for piscibactin production and uptake pathway is proposed.

## 2. Results

### 2.1. The Thioesterase Irp4 Is Required for Piscibactin Biosynthesis

**Figure 2:**
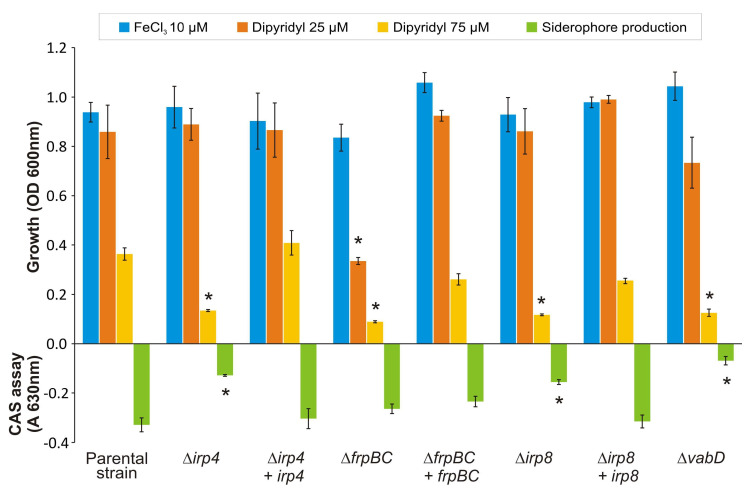
The growth of *V. anguillarum* parental strain (RV22 Δ*vabF*) and its derivative mutants under iron excess (10 µM FeCl_3_) and iron-restrictive (25–75 µM 2,2′-dipyridyl) conditions. Siderophore production was quantified by CAS liquid assay in cell-free supernatants of cultures grown in CM9 supplemented with 25 µM 2,2′-dipyridyl. Uninoculated CM9 with 25 μM 2,2′-dipyridyl was used as blank. Asterisk denotes statistically significant differences, *p* < 0.05 (student’s *t*-test).

The translated product of *irp4* showed sequence similarity (42% of identity, 56% similarity) to type II TEs such as YbtT from the NRPS/PKS biosynthetic pathway of yersiniabactin [17]. In addition, Irp4 contains the characteristic conserved TE signature motif GHSMG [18] from the amino acid position 99 to 103. This finding suggests that *irp4* encodes a functional type II TE. To analyse the role of Irp4 in piscibactin production, a defective mutant for this gene was constructed in a RV22 ∆*vabF* background, a *V. anguillarum* mutant impaired to synthetize vanchrobactin (the other siderophore produced by *V. anguillarum* RV22) and that only synthesizes piscibactin [7]. The RV22 Δ*vabD* mutant (lacking the phosphopantetheinyl transferase) impaired for siderophore synthesis (does not produce piscibactin nor vanchrobactin) was used as a non siderophore-producer control. Then, the resultant RV22 ∆*vabF*∆*irp4* mutant was challenged to grow and produce siderophores under different iron-availability conditions. As shown in Figure 2, RV22 ∆*vabF*∆*irp4* double mutant, the parental strain RV22 ∆*vabF* and RV22 ∆*vabD* were able to grow under iron excess (CM9 plus 10 µM FeCl_3_) and under mild iron restrictive conditions (CM9 with 25 µM 2,2′-dipyridyl), showing indistinguishable growth levels. By contrast, RV22 ∆*vabF*∆*irp4* mutant was impaired to grow under severe iron-restricted conditions achieved by the addition of 2,2′-dipyridyl at 75 µM.

**Figure 3:**
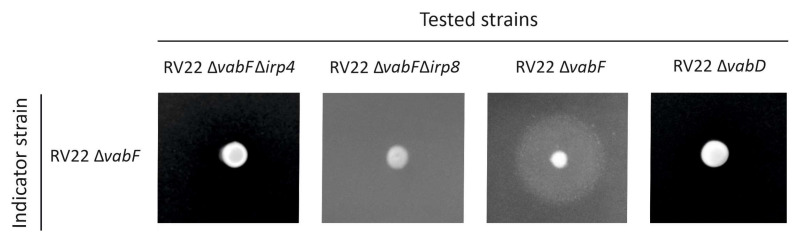
The cross-feeding assay to evaluate the ability of the tested strains (RV22 Δ*vabF*Δ*irp4*, RV22 Δ*vabF*Δ*irp8*, RV22 Δ*vabF*, and RV22 Δ*vabD*) to produce siderophores that the indicator strain (RV22 Δ*vabF*, which express the piscibactin transporter FrpA) could use as iron source to grow under iron limited conditions.

Evaluation of siderophore content by the CAS liquid assay in the cell-free supernatants showed that the deletion of *irp4* (RV22 ∆*vabF*∆*irp4*) caused a significant reduction in siderophore production compared to the parental strain RV22 ∆*vabF* (Figure 2). Siderophore production by the ∆*irp4* mutant was also tested by cross-feeding bioassays. The results showed that RV22 ∆*vabF*∆*irp4* mutant was unable to cross-feed its parental strain RV22 ∆*vabF*, the same result displayed by the non siderophore-producer RV22 ∆*vabD* (Figure 3). Thus, the growth ability under iron restriction and siderophore production phenotype of RV22 ∆*vabF*∆*irp4* and RV22 ∆*vabD* were indistinguishable. RV22 ∆*vabF*∆*irp4* mutant complemented with a functional version of the *irp4* gene recovered siderophore production and showed an identical growth phenotype to that observed for the parental strain RV22 ∆*vabF* (Figure 2). These results provide clear evidence that the putative thioesterase encoded by *irp4* is a key function required for piscibactin synthesis.

### 2.2. Irp8 Is Required for Piscibactin Secretion

The *irp*-HPI encodes a putative MFS-like exporter named Irp8 whose predicted protein structure shares the 12 transmembrane segments typically found in this type of efflux pumps [19]. To analyse the putative role of Irp8 in piscibactin exporting route, an in-frame deletion mutant of the *irp8* gene was constructed and its growth ability and siderophore production were evaluated (Figure 2). When grown in iron excess or mild iron-restrictive conditions, the mutant RV22 Δ*vabF*Δ*irp8* showed a growth ability indistinguishable from that of the parental strain RV22 Δ*vabF*. By contrast, it was unable to grow under iron limitation at 75 µM of 2,2′-dipyridyl. The decrease in the growth levels of RV22 Δ*vabF*Δ*irp8* under iron restriction correlates with a lower siderophore production (Figure 2). Nonetheless, the Δ*irp8* mutant achieved an A_630_ ca. −0.15 in the CAS liquid assay, which suggests that irp8 deletion did not totally abolish siderophore production. Several studies suggest that most siderophore exporters mutants show intermediate growth and siderophore production phenotypes when compared to the respective parental strains [19,20]. These phenotypes could be caused by the presence of alternative secretion routes or the passive secretion of subproducts of siderophore synthesis that still show some siderophore activity when analyzed by the CAS liquid assay [19,20]. Notably, the RV22 Δ*vabF*Δ*irp8* mutant was unable to cross-feed RV22 Δ*vabD* strain (Figure 3). These results greatly suggest that RV22 Δ*vabF*Δ*irp8* mutant is deficient in piscibactin production and thus Irp8 would be required to export this siderophore.

### 2.3. Mutants with Deleted Irp4 or Irp8 Genes Are Unable to Produce Piscibactin

**Figure 4:**
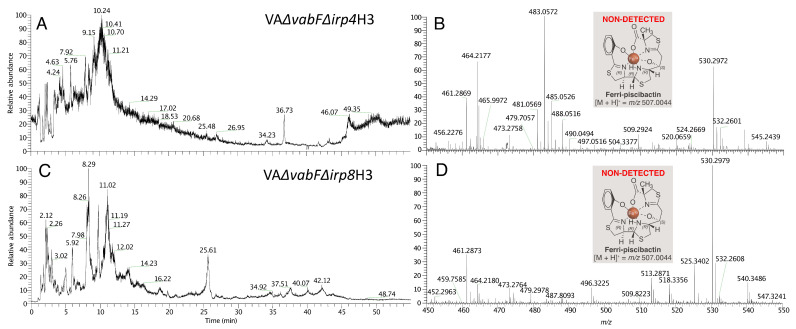
(**A**) HPLC/HRMS analysis of VA∆vabF∆irp4H3 and VA∆vabF∆irp8H3 fractions, eluted from the HLB cartridge with H_2_O/CH_3_CN (1:1), from *V. anguillarum* RV22 ∆*vabF*∆*irp4* and RV22 ∆*vabF*∆*irp8* mutant strain supernatants, respectively, for the detection of ferri-piscibactin. (**A**) Total Ion Current (TIC) chromatogram of VA∆vabF∆irp4H3 fraction. (**B**) (+)-HRESIMS of VA∆vabF∆irp4H3 for the time range from 8 to 12 min based on ferri-piscibactin reported retention time (t_R_ = 10.30 min) showing the absence of its [M + H]^+^ ion adduct (calcd. for C_19_H_21_N_3_O_4_S_3_Fe^+^ *m/z* 507.0044). (**C**) Total Ion Current (TIC) chromatogram of VA∆vabF∆irp8H3 fraction. (**D**) (+)-HRESIMS of VA∆vabF∆irp8H3 for the time range from 8 to 12 min based on ferri-piscibactin reported retention time (t_R_ = 10.30 min) showing the absence of its [M + H]^+^ ion adduct (calcd. for C_19_H_21_N_3_O_4_S_3_Fe^+^ *m/z* 507.0044).

To confirm the inability of *V. anguillarum* RV22 ∆*vabF*∆*irp4* and RV22 ∆*vabF*∆*irp8* mutants to produce piscibactin, culture supernatants of these mutants were analyzed following our SPE-HLB/HPLC-HRMS methodology [4,7] (see Materials and Methods section). Thus, the supernatants from the cultures grown under iron restriction were treated with FeCl_3_, to obtain the stable ferri-siderophores, and fractionated using HLB cartridges. The fractions eluted with H_2_O/CH_3_CN (1:1), described as the ferri-piscibactin containing fractions, VA∆vabF∆irp4H3 and VA∆vabF∆irp8H3 from the mutants RV22 ∆*vabF*∆*irp4* and RV22 ∆*vabF*∆*irp8*, respectively, were analyzed by HPLC/HRMS (Figure 4). The search of [M + H]^+^ ion adduct corresponding to ferri-piscibactin (calcd. for C_19_H_21_N_3_O_4_S_3_Fe^+^ *m/z* 507.0044) was carried out within the time range from 8 to 12 min based on its reported retention time (t_R_: 10.3 min). The analysis showed complete absence of this compound in both fractions, demonstrating the essential role of the genes irp4 and irp8 in piscibactin biosynthesis and secretion.

### 2.4. Inactivation of FrpBC Disables Ferri-Piscibactin Utilization as Iron Source

**Figure 5:**
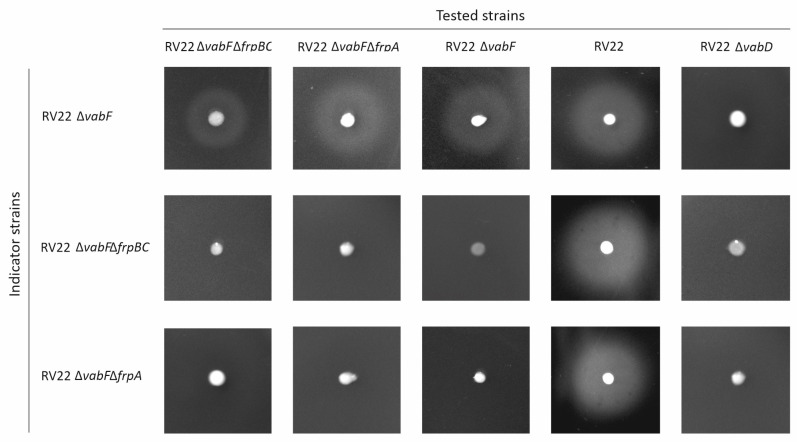
Cross-feeding assay to evaluate piscibactin production by the tested strains (RV22, RV22 Δ*vabD*, RV22 Δ*vabF*, RV22 Δ*vabF*Δ*frpBC* and RV22 Δ*vabF*Δ*frpA*) and its use by the indicator strains (RV22 Δ*vabF*, RV22 Δ*vabF*Δ*frpA* and RV22 Δ*vabF*Δ*frpBC*). A growth halo of the indicator strains around the tested strains indicates that they can use the siderophore produced by the tested strains to grow under iron limited conditions.

Genes *frpBC* were proposed to encode a probable ABC transporter involved in ferri-piscibactin transport through the inner membrane [5,7]. To demonstrate the role of FrpBC in piscibactin uptake, an in-frame deletion mutant of both *frpB* and *frpC* genes was constructed in a RV22 Δ*vabF* (impaired for vanchrobactin synthesis) background. Then, mutant and parental strains were grown in CM9 minimal medium under different iron availability conditions (Figure 2). When grown under iron excess or under weak iron restriction, parental strain RV22 Δ*vabF* and its derivative RV22 Δ*vabF*Δ*frpBC* mutant showed indistinguishable growth capacities (Figure 2). However, this mutant showed a severe defect in its growth ability at 75 µM 2,2′-dipyridyl (Figure 2). This reduced growth ability contrasts with its siderophore production since RV22 Δ*vabF*Δ*frpBC* mutant showed siderophore production levels (by the CAS assay) similar to the parental strain (Figure 2). These results greatly suggest that piscibactin is being synthetized and exported, but it is not being used as an iron source. The cross-feeding assays showed that RV22 Δ*vabF*Δ*frpBC* can cross-feed its parental strain (Figure 5). By contrast, it does not support the growth of RV22 Δ*vabF*Δ*frpA* mutant (Figure 5), a strain unable to use piscibactin as it lacks the TBDT FrpA [16]. Conversely, RV22 Δ*vabF*Δ*frpBC* cannot grow when cross-fed by piscibactin producing strains, either the parental RV22 Δ*vabF* strain or itself (RV22 Δ*vabF*Δ*frpBC*). However, it can be cross-fed by the wild-type strain RV22 due to the production of vanchrobactin (Figure 5). These results suggest that, although piscibactin production and secretion is accomplished in RV22 Δ*vabF*Δ*frpBC*, ferri-piscibactin internalization is impaired, preventing the use of ferri-piscibactin as an iron source. Thus, FrpBC would be required for the internalization of the ferri-piscibactin complex.

### 2.5. Transcriptional Regulation of FrpBC Genes

**Figure 6:**
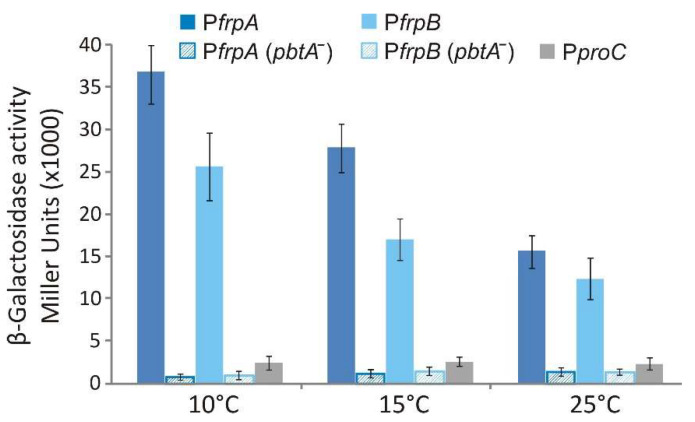
The transcriptional activity of promoters P*frpA* and P*frpB* at three different temperatures (10, 15 and 25 °C) measured in a *V. anguillarum* RV22 Δ*vabF* background (solid color bars) and in a *V. anguillarum* RV22 Δ*vabF*Δ*pbtA* background (striped bars). Constitutive promoter P*proC* was used as control.

Genes *frpBC* are located downstream of the large operon *frpAirp8213495* but expressed from the opposite DNA strand (Figure 1b). Since *frpBC* open reading frames are concatenated, their expression must be controlled by a promoter located upstream of *frpB* (Figure 1b). To identify the *frpB* promoter region and study its expression pattern, the 760 bp immediately upstream of the *frpB* start codon were fused to a promoterless *lacZ* and its transcriptional activity evaluated under low iron conditions (25 µM 2,2′-dipyridyl) at 10, 15 and 25 °C (Figure 6). The results show that the region upstream of *frpB* contains an active promoter since significative β-galactosidase activity was measured in the *V. anguillarum* RV22 Δ*vabF* background. More notably, as occurs with P*frpA*, P*frpB* expression activities were inversely proportional to the growth temperature, showing the maximum transcription level at 10 °C, the lowest temperature assayed. Finally, when P*frpA* and P*frpB* expression activity was analyzed in a *V. anguillarum* mutant defective in the transcriptional regulator PbtA (RV22 Δ*vabF*Δ*pbtA*) [11], the transcriptional activity of both promoters was completely abolished (Figure 6).

## 3. Discussion

**Figure 7:**
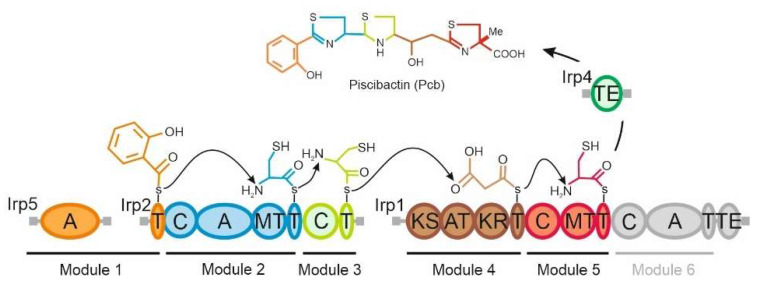
A biosynthetic model for piscibactin assembly showing module organization and predicted substrate of Irp5, Irp2 and Irp1. Conserved domain annotation: A—adenylation, T—aryl or peptidyl carrier protein, C—condensation, MT—methyltransferase, KS—ketoacyl synthase, AT—acyltransferase, KR—ketoacyl reductase, TE—thioesterase.

Functions related to the biosynthesis and transport of siderophores are attractive targets to develop new antibacterial compounds [21,22]. However, the establishment of the precise role of proteins related to siderophore biosynthesis and transport is mandatory to rationally design antibacterials targeting siderophore systems. The piscibactin system present in the *Vibrionaceae* family possesses a C-terminal TE domain as part of the NRPS/PKS enzyme Irp1 in addition to a separate gene (*irp4*) that encodes a putative external TE (Figure 7). Gene *irp4* is located between the biosynthetic genes *irp3* and *irp5* with which it is co-transcribed [5,7]. External TEs are generally related to editing functions, as they can remove non-elongatable structures to promote the continuous flow of several rounds of biosynthesis by liberating the CP domains of NRPS/PKS from precursors [14]. Piscibactin biosynthetic pathway is constituted by the NRPS/PKSs Irp5, Irp2 and Irp1, which form a synthesis complex organized in 6 modules (Figure 7). Irp1 contains an internal C-terminal thioesterase domain that would release the complete siderophore at the final step of biosynthesis (Irp1 module 6). However, the predicted final product of the route was not detected either in *P. damselae* subsp. *piscicida* or in *V. anguillarum* supernatants [4,7], and the piscibactin chemical structure is in accordance with the early release of the nascent siderophore at domain 5 (Figure 7). Since *irp4* defective mutant lacks siderophore production, the results greatly suggest that Irp4 is required for piscibactin production since it mediates the early liberation of the siderophore from Irp1. The yersiniabactin system also possesses a C-terminal TE domain as part of the NRPS/PKS synthetase HMWP1 and an external TE named YbtT [23]. Both TE domains are functional and ensure appropriate levels of yersiniabactin production [24]. While the internal C-terminal TE releases the complete siderophore from the multienzymatic complex, YbtT avoids the formation of aberrant molecules that would block siderophore synthesis [24]. Thus, although YbtT is not required for bacterial growth, it is needed for yersiniabactin maximal production as it prevents the incorporation of erroneous precursors that inhibit the pathway [25]. The inability to detect the predicted final siderophore structure of the piscibactin assembly line questions the role of the Irp1 C-terminal TE domain. It cannot be ruled out that module 6 is not functional or that its product may be a cryptic metabolite that is synthesized only under specific conditions [4]. Nonetheless, the loss of Irp4 activity abolishes siderophore production, denoting a direct role of Irp4 in piscibactin synthesis and in its release from the NRPS/PKS multienzymatic system.

Secretion of secondary metabolites requires at least three components: an active efflux pump, a membrane fusion protein that connects the pump to the outer membrane, and a channel located at the outer membrane that allows the passage of the siderophore [19]. Although mechanisms behind the secretion of siderophores remain uncharacterized in most bacteria, the two major export systems that are usually involved in this process belong to ATP-dependent efflux pumps [26] and MFS-like transporters [27]. The enterobactin siderophore export system in *E. coli* is one of the best characterized secretion routes [19,28]. Enterobactin is exported to the periplasm through the MFS-like transporter EntS [19]. Then, it is captured by resistance–nodulation–cell division (RND) family proteins AcrB, AcrD and MdtABC, and exported to the extracellular environment through the outer membrane channel TolC [28,29]. Both RND efflux systems and TolC channels are ubiquitous transporters and exhibit a broad substrate specificity [30,31]. Interestingly, the involvement of a RND efflux system has been described for the secretion of siderophores in some *Vibrionaceae* members, such as vibriobactin of *V. cholerae* [32] and vulnibactin of *V. vulnificus* [33]. VabS was previously characterized in *V. anguillarum* as the MFS-like transporter essential for the secretion of the siderophore vanchrobactin [20]. The results described here show that the MFS efflux pump Irp8, encoded by the *irp*-HPI element, is required for piscibactin secretion. All the results put together suggest that, once in the periplasm, yet uncharacterized RND family protein(s) coupled to the outer membrane channel TolC-like must complete the secretion of both siderophores, piscibactin and vanchrobactin, to the extracellular environment [34].

Special emphasis is currently being focused on functions required for ferri-siderophore uptake since they can be used to vectorize antimicrobial compounds following the Trojan-horse strategy [35]. The ferri-siderophore import occurs in a stepwise manner. Firstly, it must pass the outer membrane through a specific TBDT. Once the complex is at the periplasm, it is usually combined with a periplasmic binding protein and then passes the inner membrane through the ABC transporter to be delivered at the cytoplasm [34]. Acquisition of *irp*-HPI confers the ability to produce and use piscibactin as an iron source, but it does not contain candidate genes to encode a putative ferri-piscibactin periplasmic binding protein [6]. Moreover, some ferri-siderophores complexes can be dissociated in the periplasm and the reduced iron internalized via the Feo system [36]. Thus, different iron release strategies are observed in the cytoplasm or periplasm depending on the siderophore fate [37]. The dramatic decrease in the ability to grow under weak iron restrictive conditions and the inability to be cross-fed by piscibactin producing strains showed that FrpBC are required for the internalization of ferri-piscibactin. Consequently, our results greatly suggest that the import of ferri-piscibactin into the cytoplasm is required for the release of iron and its subsequent incorporation into the bacterial metabolism. FrpA was recently characterized as the ferri-piscibactin TBDT, and we have demonstrated that some synthetic piscibactin mimics are also transported through FrpA and internalized in *V. anguillarum* [16]. These siderophore mimics could be further used as antimicrobial vectors using the Trojan-horse strategy. Definition of the cell compartment where the ferri-siderophore is released has great importance in selecting an appropriate antibiotic to be used as cargo in a Trojan-horse strategy [22].

The sequence upstream of *frpA* (P*frpA*) controls the expression of a large operon that includes the ferri-piscibactin TBDT FrpA; the siderophore exporter Irp8; and the biosynthetic functions Irp123459 (Figure 1b) [7]. P*frpA* transcription level is up-regulated below the optimum growth temperature (>20 °C) under iron starvation and depends on the transcriptional activator PbtA to be active [7,11,38]. Current results showed that the sequence immediately upstream of *frpB* is the promoter that controls the expression of the ABC transporter FrpBC. Interestingly, P*frpB* transcriptional activity is also temperature-dependent and requires the transcriptional activator PbtA to be active. All these findings together show that P*frpA* and P*frpB* are the main promoters that control the expression of piscibactin synthesis and transport genes. Notably, since both promoters are up-regulated under low-iron availability and cold temperature, expression of genes encoding the ferri-piscibactin uptake system FrpABC is coordinated with the expression of genes encoding the biosynthesis functions.

**Figure 8:**
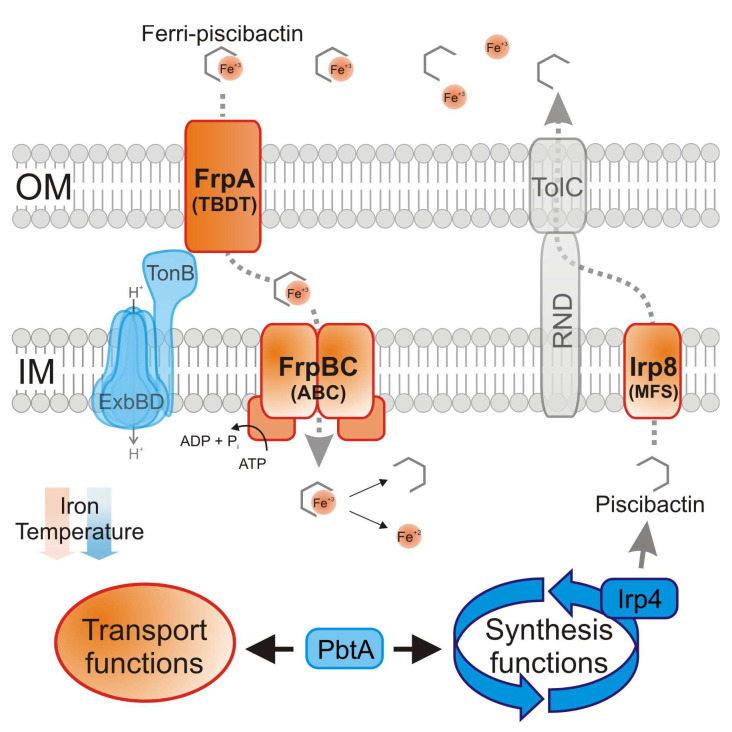
A model of production and utilization of piscibactin in *V. anguillarum*. Piscibactin genes are induced when *V. anguillarum* grows under low-iron conditions at cold temperature. The transcriptional activator PbtA is required to express both biosynthesis and transport functions. During synthesis, the thioesterase Irp4 early releases nascent piscibactin from the NRPS/PKS enzyme line. After synthesis, piscibactin is exported to the extracellular space through the MFS protein Irp8. RND proteins and the outer membrane channel TolC would likely participate in the secretion process. Once the ferri-piscibactin complex is formed in the external medium, it would be internalized to the cytoplasm through the outer membrane TBDT FrpA and ABC transporter FrpBC. Final iron release from the ferri-piscibactin complex and its further incorporation to the cell metabolism is accomplished in the cytoplasm. OM, outer membrane; IM, inner membrane; RND, resistance-nodulation-cell division protein; TBDT, TonB-dependent transporter; ABC, ATP-binding cassette transporter.

Based on the results described in this work, together with previous findings, we can propose here a model for piscibactin production and the utilization pathway (Figure 8). The thioesterase Irp4 is required to synthesize piscibactin, so it mediates the release of nascent piscibactin at module 5 of the NRPS Irp1 (Figure 7 and Figure 8). Once synthesized, piscibactin is exported to the extracellular medium through the MFS Irp8 (Figure 8), which would be coupled to RND family efflux systems [34]. Once formed, the ferri-piscibactin complex, it would be acquired through the outer membrane TBDT FrpA and ABC transporter FrpBC. Our results greatly suggest that ferri-piscibactin must reach the bacterial cytoplasm to complete the release of iron and its further incorporation into the bacterial metabolism (Figure 8). The results described here could be used to design future therapeutic strategies targeting the piscibactin system, e.g., sideropohore-antibiotic conjugates based on piscibactin mimics.

## 4. Materials and Methods

### 4.1. Bacterial Strains, Plasmids and Media

Bacterial strains and plasmids used in this work are listed in Table 1. *V. anguillarum* strains were grown at 25 °C or 15 °C in Tryptic Soy Broth (TSB-1) or Tryptic Soy Agar (TSA-1) (Condalab, Madrid, Spain) supplemented until 1% NaCl. *Escherichia coli* strains were grown at 37 °C in Luria Bertani (LB) Broth or Agar (Condalab, Madrid, Spain). When required, antibiotics were added at the following final concentrations: ampicillin sodium salt 100 µg mL^−1^ or 60 µg mL^−1^, kanamycin 50 µg mL^−1^ and gentamycin 15 µg mL^−1^.

### 4.2. Construction of Irp4, Irp8 and FrpBC Defective Mutants and Mutants Reversion

In-frame deletion mutants of *irp4*, *irp8* and *frpBC* genes were constructed by allelic exchange in a *V. anguillarum* RV22 Δ*vabF* background (unable to synthesize vanchrobactin) as previously described [20]. Briefly, deleted alleles of each gene were constructed by PCR amplification of the flanking regions of each gene and subsequent cloning of both regions into the low-copy number plasmid pWKS30 [42]. Then, deleted alleles were liberated by digestion with *Not*I and *Apa*I and cloned into the suicide vector pCAR109 [43]. The plasmid was then mobilized to RV22Δ*vabF* strain by conjugation and the transconjugants were selected based on ampicillin and kanamycin resistance. After a second event of recombination and consecutive passages under no selective pressure, the mutant strains were selected based on sucrose resistance (15%). The loss of the plasmid was confirmed by screening bacterial growth on kanamycin and ampicillin plates. The allelic exchange event was confirmed by PCR and Sanger sequencing. Primers used in this work are shown in Table 2.

Reversion to parental alleles of *irp4*, *irp8* and *frpBC* defective mutants was accomplished through the reintroduction of the wild type gene(s) by allelic exchange. To this purpose the complete wild type gene(s) and flanking regions were PCR amplified and cloned in pCAR109. The reintroduction of wild type genes, selection of both recombination events and final confirmation of the process was carried out as previously detailed for mutant construction.

### 4.3. Growth Ability and Siderophore Production Assays

Growth ability assays were performed in 5 mL of CM9 minimal medium [39] supplemented with 10 µM FeCl_3_ to achieve iron excess conditions or with the iron chelator 2,2′-dipyridyl at 25 and 75 μM, to achieve iron restricted conditions. As inoculum was used a 1:50 dilution of a *V. anguillarum* overnight culture grown in TSB-1 to an OD_600_ = 0.5. After 48 h of incubation at 15 °C with shaking at 150 rpm, growth achieved (OD_600_) was recorded in a spectrophotometer (Hitachi, Tokyo, Japan). Bacterial cultures grown in CM9 supplemented with 25 μM 2,2′-dipyridyl (OD_600_ ≈ 0.8) were used to obtain supernatants and measure siderophore production using the chrome azurol-S (CAS) liquid assay [45]. For this purpose, equal volumes of cell free supernatants and CAS reagent were mixed and, after 15 min of incubation at room temperature, A_630_ was measured in a spectrophotometer (Hitachi, Tokyo, Japan). Uninoculated CM9 with 25 μM 2,2′-dipyridyl was used as blank in all spectrophotometric measures for siderophores quantification.

### 4.4. Cross-Feeding Assays

The ability to produce or use piscibactin was determined via cross-feeding experiments. To test whether *V. anguillarum irp4* (RV22 Δ*vabF*Δ*irp4*), *irp8* (RV22 Δ*vabF*Δ*irp8*) and *frpBC* (RV22 Δ*vabF*Δ*frpBC*) defective mutants produce piscibactin, a cross-feeding assay was conducted using *V. anguillarum* RV22 Δ*vabF* as indicator strain as it uses piscibactin as an iron source. Indicator strains were inoculated into CM9 plates as follows: 0.5 mL of an overnight culture in TSB-1 at an OD_600_ = 0.5 were mixed with 20 mL of CM9 medium containing 0.8% agarose and 2,2′-dipyridyl 100 µM, a concentration close to the minimal inhibitory concentration (MIC) and at which growth halos can be easily visualized [20]. The strains to be tested were cultured in TSA-1 plates supplemented with 50 μM 2,2′-dipyridyl and the cells were harvested with a sterile loop and placed onto the surface of the plates previously inoculated with the indicator strains. To test whether *V. anguillarum frpBC* defective mutant could use ferri-piscibactin as an iron source, it was also used as indicator strain. The presence of growth halos of the *V. anguillarum* indicator strains around cells of *V. anguillarum* after 48 h incubation at 15 °C was indicative of piscibactin production. *V. anguillarum* RV22 wild type strain (piscibactin and vanchrobactin producer), RV22 Δ*vabF* (piscibactin producer) and RV22 Δ*vabF*Δ*frpA* (piscibactin producer but impaired to use ferri-piscibactin as iron source) were used as controls.

### 4.5. LacZ Transcriptional Fusions and β-Galactosidase Assays

The probable promoter of *frpBC* genes was PCR amplified and fused to a promoterless *lacZ* gene in the low-copy-number reporter plasmid pHRP309 [44]. The PCR-amplified region was a fragment of about 700 bp, including the first nucleotides of the *frpB* coding sequence (ca. 50 bp) and the region upstream of the start codon. The resulting transcriptional fusion construct, *frpB::lacZ* (pML212), was mobilized from *E. coli* S17-1 λ*pir* to *V. anguillarum* by conjugation. The previously obtained promoter fusion *frpA::lacZ* (pMB276) [7] was also evaluated. The *V. anguillarum* Δ*vabF* and Δ*pbtA* mutant strains carrying one of the promoter–*lacZ* fusions: *frpB::lacZ* (pM212), *frpA::lacZ* (pMB276) or the plasmid pHRP309 alone (negative control) were grown in CM9 minimal medium under low iron conditions (25 µM 2,2′-dipyridyl) at 10, 15 and 25 °C. When cultures achieved a OD_600_ ca. 0.3, the *β*-galactosidase (LacZ) activity of each culture was measured by the method of Miller [46]. Results shown are the means of three independent experiments.

### 4.6. Analysis of the Presence of Piscibactin by SPE-HLB/HPLC-HRMS

The presence of piscibactin in supernatants was studied following the SPE-HLB/HPLC-HRMS methodology described by our research group [4,7]. Briefly, mutant strains *V. anguillarum* RV22 ∆*vabF*∆*irp4* and RV22 ∆*vabF*∆*irp8* were grown at 15 °C under iron-deprived conditions (30 µM 2,2′-dipyridyl) until an OD_600_ = 1. The cultures were then centrifuged at 4,000 rpm for 30 min (Beckman J-21 High Speed Centrifuge) and filtrated through a 0.45 µm pore size membrane. The resultant cell-free supernatants (1 L) were concentrated under reduced pressure conditions to a volume of 300 mL, treated with FeCl_3_ (19 mg) for 5 min and incubated at 4 °C overnight. The resultant solutions were fractionated in three batches of 75 mL by Oasis^®^ Hydrophilic Lipophilic Balance (HLB) cartridges (35 cm^3^, 6 g, Waters), previously conditioned with 60 mL of CH_3_CN (solvent B) followed by 60 mL of deionized H_2_O (solvent A). The batches were fractionated with 0:1, 3:1, 1:1, 1:3, and 0:1 of A/B (30 mL) to afford the fractions VA∆vabF∆irp4H1-H5 and VA∆vabF∆irp8H1-H5 of the mutant strains *V. anguillarum* RV22 ∆*vabF*∆*irp4* and RV22 ∆*vabF*∆*irp8*, respectively. Fractions eluted with 1:1 of A/B, VA∆vabF∆irp4H3 and VA∆vabF∆irp8H3, were analyzed by HPLC/HRMS using an Atlantis^®^ C18 column (100 × 4.6 mm, 5 μm) (Waters) in a HPLC Accela (Thermo) coupled to an LQT-Orbitrap Discovery mass spectrometer and a PDA detector. The HPLC method consisted of the following gradient steps (solvent A: H_2_O, solvent B: CH_3_CN): 35 min from 10% to 100% of B, an isocratic step of 10 min at 100% of B, 10 min from 100 to 10% of B and a final isocratic step of 10 min at 10% B, using a flow rate at 1 mL min^−1^. Mass data were acquired in full positive scan mode using a collision energy (CE) of 35 eV and a capillary temperature of 350 °C.

## Figures and Tables

**Table 1:** The bacterial strains and plasmids used in this work.

Strains	Relevant Characteristics	Source
*V. anguillarum*		
RV22	Wild-type serotype O2 strain isolated from diseased turbot (Spain)	[39]
MB14	RV22 with in-frame deletion of *vabF* gene	[20]
MB67	RV22 with in-frame deletion of *vabD* gene	[40]
ML178	MB14 with in-frame deletion of *irp4* gene	This study
ML772	MB14 with in-frame deletion of *irp8* gene	This study
ML886	MB14 with in-frame deletion of *frpBC* genes	This study
ML575	ML178 revertant strain by the reintroduction of *irp4* wild type gene	This study
ML955	ML886 revertant strain by the reintroduction of *frpBC* wild type genes	This study
ML979	ML772 *irp8* genes revertant to the *irp8* parental phenotype	This study
	*E. coli*	
DH5α	Cloning strain	Laboratory strain
S17-1- *λpir*	RP4 (Km::Tn7, Tc::Mu-1) *pro-82 λpir recA1 end A1 thiE1 hsdR17 creC510*	[41]
Plasmids		
pWKS30	Low-copy number cloning vector	[42]
pNidKan	Suicide vector derived from pCVD441	[43]
pHRP309	Low-copy number *lacZ* reporter plasmid, *mob* Gm^r^	[44]
pMB276	*frpA* promoter (P*frpA*) fused to the promoterless *lacZ* gene in pHRP309	[40]
pML212	*frpBC* promoter (P*frpBC*) fused to the promoterless *lacZ* gene in pHRP309	This study

**Table 2:** The primers used in this work.

Oligonucleotide	Sequence (5’ -> 3’) ^a^	Size (bp)
*irp4* mutant construction		
1_Irp4ang_XbaI	CGCTCTAGAGTCTCATTGCAAATGCGCCA	723
2_Irp4ang_PstI	CGCCTGCAGGGCACCATTCTGATAAAGTG	
3_Irp4ang_PstI	CGCCTGCAGCTGATAGCCATATCAGGCGA	861
4_Irp4ang_XhoI	CCGCTCGAGAGCTTGAGCATGAAAGAGCG	
*irp8* mutant construction		
1_Irp8_F_XbaI	GGCTCTAGATCGCTTAGCTGACAACATGG	824
2_Irp8_R_BamHI	CGCGGATCCAGTGATGCCCTGTTGTCGAA	
3_Irp8_F_BamHI	CCGGGATCCCTGCTGACATTCTCCGTTAC	819
4_Irp8_R_XhoI	GCCCTCGAGCATGGCTTGTTCAGCGTCAT	
*frpBC* mutant construction		
1_FrpBC_R_NotI	CCGGCGGCCGCTCTCAGCACGTGGAAAGCGA	1320
2_FrpBC_F_PstI	GGCCTGCAGGCTGCGCAGTTTATCCATTC	
3_FrpBC_R_PstI	CCGCTGCAGGCGCCTATCTTACTGCTTGA	995
4_FrpBC_F_KpnI	GCCGGTACCGACCAATATCTCACCGTGAC	
*irp4* complementation		
Irp4_comp_F_NotI	CCGGCGGCCGCGTCCAATACCGAGTCAACAG	2511
Irp4_comp_R_ApaI	GCGGGGCCCAGCGGCATGTTCGGCAATTT	
*irp8* complementation		
Irp8_comp_F_NotI	CCGGCGGCCGCTCGCTTAGCTGACAACATGG	2558
Irp8_comp_R_ApaI	GCCGGGCCCCATGGCTTGTTCAGCGTCAT	
*frpBC* complementation		
FrpBC_comp_F_ApaI	GCCGGGCCCGACCAATATCTCACCGTGAC	5062
1_FrpBC_R_NotI	CCGGCGGCCGCTCTCAGCACGTGGAAAGCGA	
P*frpB* promoter fusion construction		
Transp_F_BamHI	CCGGGATCCCGATAAGGTGACGCGATTTC	746
Transp_R_XbaI	CGCTCTAGAAGCGGATGGTCAAGACTTTG	

^a^ Restriction sites used are underlined.

## Data Availability

Not applicable.
